# Prevalence, Colonization, Epidemiology, and Public Health Significance of *Clostridioides difficile* in Companion Animals

**DOI:** 10.3389/fvets.2020.512551

**Published:** 2020-09-18

**Authors:** Belen G. Hernandez, Akhil A. Vinithakumari, Brett Sponseller, Chandra Tangudu, Shankumar Mooyottu

**Affiliations:** ^1^Department of Veterinary Pathology, Iowa State University, Ames, IA, United States; ^2^Department of Veterinary Microbiology and Preventive Medicine, Iowa State University, Ames, IA, United States

**Keywords:** companion animals, *Clostridioides difficile*, prevalence, molecular epidemiology, public health

## Abstract

*Clostridioides difficile*, previously *Clostrdium difficile*, is a major cause of antibiotic-associated enteric disease in humans in hospital settings. Increased incidence of *C. difficile* infection (CDI) in community settings raises concerns over an alternative source of CDI for humans. The detection of genetically similar and toxigenic *C. difficile* isolates in companion animals, including asymptomatic pets, suggests the potential role of household pets as a source of community-associated CDI. The close association between companion animals and humans, in addition to the use of similar antibiotics in both species, could provide a selective advantage for the emergence of new *C. difficile* strains and thus increase the incidental transmission of CDI to humans. Therefore, screening household pets for *C. difficile* is becoming increasingly important from a public health standpoint and may become a part of routine testing in the future, for the benefit of susceptible or infected individuals within a household. In this review, we analyze available information on prevalence, pathophysiology, epidemiology, and molecular genetics of *C. difficile* infection, focusing on companion animals and evaluate the risk of pet-borne transmission of CDI as an emerging public health concern. Molecular epidemiological characterization of companion animal *C. difficile* strains could provide further insights into the interspecies transmission of CDI. The mosaic nature of *C. difficile* genomes and their susceptibility to horizontal gene transfer may facilitate the inter-mixing of genetic material, which could increase the possibility of the emergence of new community-associated CDI strains. However, detailed genome-wide characterization and comparative genome analysis are warranted to confirm this hypothesis.

## Introduction

*Clostridioides difficile* is an anaerobic spore-forming bacterium that causes a serious toxin-mediated enteric disease in humans (1). Annually, nearly half a million people in the United States suffer from *C. difficile* infection (CDI) (2), which incurs ~6.3 billion dollars of treatment and other hospital costs (3). Relapse of CDI usually occurs in ~20% of the individuals within a month after primary treatment (4, 5). Currently, there are no definitive treatment options available for CDI without the possibility of recurrence or relapse (5). A recent study indicated that 1 out of every 11 patients with CDI died within 30 days of diagnosis (2). *C. difficile* is classically considered a nosocomial pathogen and a major cause of antibiotic-associated diarrhea in hospitalized patients. However, an increase in the number and severity of CDI in humans has been reported outside the hospital environment or in individuals with onset of symptoms 48 h or less after hospital admission, referred to as community-associated infections (6). A paradigm shift has been observed in the CDI epidemiology in recent years and the incidence rate of community-acquired *C. difficile* infections is over 40% of the total CDI cases reported (2, 7). Moreover, newer reports indicate that the national burden of nosocomial *C. difficile* infection in the United States has decreased by 36%, whereas community-associated *C. difficile* infection burden has shown no change in trend (8). Notably, a definitive source of *C. difficile* in community settings has not been identified so far.

*Clostridioides difficile* has been isolated repeatedly from the intestinal flora of healthy domesticated animals, including pets, and associated with the sporadic incidence of diarrhea in susceptible animals (9–11). An increase in the isolation of *C. difficile* from food-animals and animal derived food has been attributed to the increased reports of community-associated human CDI (12). In the past decade, several investigators have isolated and characterized food-animal and meat strains of *C. difficile*. As an example, a common *C. difficile* strain isolated in pigs, ribotype (RT) 078, is also a ribotype commonly implicated in human community-associated *C. difficile* infection (13). However, other studies have questioned the potential foodborne transmission of *C. difficile* in humans specifically due to the lack of evidence of direct transmission and low prevalence of *C. difficile* in animal-derived foods (14–16). Therefore, the search for a potential source of C. *difficile* has recently been focused on companion animals (17). The general public is more intimately associated with pets than food animals, suggesting that *C. difficile* carriage in pets, especially dogs and cats, poses a relatively high public health risk to humans in household settings.

Reports from various parts of the world suggest household pets are carriers and sources of pathogenic *C. difficile* to humans. Studies conducted in past years reported an ~4–30 percent prevalence of *C. difficile* in dogs with several toxigenic isolates, where the toxigenic strains represented nearly 50% in some instances (17–20). Furthermore, *C. difficile* ribotype RT 106 has now surpassed the hospital-acquired *C. difficile* RT 027 in becoming the most common ribotype implicated in human CDI in the United States and has been frequently isolated from dogs and cats (19, 21–25). Therefore, screening household pets for *C. difficile* is becoming increasingly important from a public health point of view and could become routine in the future. In this review, we analyze available information on colonization, pathogenesis, and epidemiology of *C. difficile* in companion animals, particularly in pets, and examine the potential pet-borne transmission to humans as an emerging public health concern.

## *C. difficile* Colonization in Dogs and Cats

Clostridial species are normal members of the intestinal flora in domestic animal species (26). Several studies indicate varying prevalence of *C. difficile* in healthy domestic animals with no enteric symptoms (27, 28). Alterations in the enteric microenvironment due to factors like antibiotic treatments, pancreatic exocrine dysfunction, changes in diet, trypsin inhibitors, poor intestinal motility or parasitic infections facilitate overgrowth of *C. difficile* (26, 29). The stress on the bacteria and overpopulation of the vegetative *C. difficile* cells triggers sporulation and synchronous secretion of potent exotoxins, toxin A (TcdA) and toxin B (TcdB) (26, 30). The toxins are endocytosed, cleaved, and release the glucosyltransferase domains into the cytosol which inactivate Rho GTPases (30, 31). Inactivation of Rho GTPases causes disruption of the cytoskeleton and intercellular tight junctions, simultaneously stimulating the intestinal epithelial and immune cells to secrete massive amounts of cytokines and chemokines (32, 33), resulting in neutrophilic inflammation and mucosal necrosis (26).

In adult dogs, colonization of toxigenic *C. difficile* in the gut is predominantly non-clinical and asymptomatic. For example, *C. difficile* toxins A, B, or combinations of both have been detected in feces of <20% of outpatient and in-patient healthy dogs as well as in-patient diarrheic dogs (27, 34). Conversely, ~90% of puppies had *C. difficile* isolated from their feces at least once during the first 10 weeks of life, of which more than half of the isolates were toxigenic (35, 36). Carriage of *C. difficile* in healthy puppies 3 months of age and older is observed to be much lower (35). The carriage rate of *C. difficile* in cats does not appear to differ from that of dogs (37) although systematic studies on *C. difficile* cat carriage are limited, in spite of litter boxes thought as a potential additional risk factor for *C. difficile* transmission within a household.

The pathogenesis and clinical features of CDI in companion animals appear to be strikingly different from that of human CDI. Gut dysbiosis is not a significant feature of CDI in dogs (26, 38), despite being a major factor in the pathogenesis of CDI in humans. Clinical signs such as acute hemorrhagic diarrhea in *C. difficile* infected dogs do not significantly correlate with the presence of *C. difficile* in the gut (27, 39). In addition, in the dysbiotic state, dogs tend to show symptoms associated with overgrowth of other cohabitating intestinal bacteria instead of a *C. difficile* toxin-mediated pathology (40). One case report indicates that cats may present with acute clinical signs of vomiting from CDI (41). Other reported clinical manifestations in cats included gas distension of the small intestines and necrotizing hemorrhagic enterotyphlocolitis (41).

Lack of concrete correlation between gut-dysbiosis and CDI in dogs provides insight into the asymptomatic carriage of *C. difficile* and plausible resistance to clinical CDI in pets. Additionally, the absence of dysbiosis suggests other potential causes or predisposing factors for CDI. Comparative microbiome analysis revealed a marked increase in the abundance of *Fusobacteria, Proteobacteria*, and *Firmicutes*, and a decrease in *Verrucomicrobia, Bacteroidetes, Euryarchaeota*, and *Actinobacteria* in *C. difficile*-carrying dogs, whereas, in humans infected with *C. difficile*, decreases in the abundance of *Firmicutes, Actinobacteria*, and *Euryarchaeota* were reported (38). Therefore, the abundance of *Firmicutes* could be a significant factor potentially associated with a lack of clinical symptoms in *C. difficile* positive dogs with dysbiosis (38). Notably, Clostridial and *Eubacteria* species, part of the *Firmicutes* phylum, possess the ability to convert primary bile acids into secondary bile acids predominantly by 7α-dehydroxylation (42). In humans, 7α-dehydroxylating bacteria increases the level of secondary bile acids, generating an intestinal bile acid profile that is associated with CDI resistance (42). Therefore, such connections should be further explored in dogs and other household pets.

Diet and gut-microbiome play a crucial role in defining the intestinal bile acid profile, thereby directly or indirectly influencing *C. difficile* colonization and infection in the host gut. In fact, distinct Clostridial species such as *Clostridium hiranonis*, with demonstrated 7α-dehydroxylating ability, were isolated from the intestines of dogs (38). *Clostridial scindens* appears to have a beneficial role in mouse models as its abundance correlates with CDI resistance (42, 43). In pet dogs, increases in relative abundance of *C. hiranonis* have been observed in the gut microbiota of the dogs fed high-intake boiled minced beef compared to dogs fed commercial dry diet (44). This change in microbiome correlated with high levels of secondary bile acids such as deoxycholic acid and ursodeoxycholic acid in the gut (44). Experimentally, *C. scindens* has previously shown resistance against CDI in an intestinal *ex-vivo* model when 7α-dehydroxylation is reconstituted to normalize bile acid composition (43). Collectively, these observations suggest a contributory role of commercial pet diet in gut-colonization of *C. difficile* in dogs. Specifically, dietary changes that promote the growth of 7α-dehydroxylating bacteria in the gut may reduce *C. difficile* carriage in pets, and thus mitigate potential zoonotic transmission of CDI. A few studies have identified the presence of *C. difficile*, occasionally toxigenic strains, in raw pet foods, suggesting an increased risk of *C. difficile* colonization in dogs and cats fed with such diets (45–47). Therefore, further investigations are required to evaluate and address the impact of contaminated pet foods on gut colonization of *C. difficile* (45).

Although clinical CDI is not well-defined in dogs, antibiotics have been used as a treatment option for enteric clostridial infections in dogs (48). Theoretically, the use of antibiotics against CDI or other disease conditions may cause the emergence of antibiotic-resistant strains of *C. difficile* within the canine gastrointestinal tract, which could be an added threat in terms of zoonotic transmission of CDI. Although the role of gut-dysbiosis has been described differently in pet CDI pathogenesis, treatments to alleviate dysbiosis have gained favor in efforts to prevent symptoms in pets and humans (49, 50). Since transmission of antibiotic-resistant *C. difficile* from companion animals appears to be a legitimate concern, antibiotic use in household pets should be revisited to prevent the emergence of antibiotic-resistant *C. difficile* strains in community settings.

## Prevalence and Major Subtypes of *C. difficile* in Companion Animals

The role of companion animals as a source for human CDI is an emerging public health concern. The lack of association between *C. difficile* colonization and clinical disease in pets allows for them to be ideal silent reservoirs of toxigenic *C. difficile* strains. Therefore, prevalence studies on *C. difficile* carriage rates in household pets are gaining more attention in the public health and medical community. Various studies have isolated toxigenic *C. difficile* strains at varying prevalence rates in dog and cat feces around the world (Table 1).

**Table 1:** Prevalence of *Clostridioides difficile* in dogs and cats.

**Location**	**No. of samples**	**Prevalence %**	**Source**
England	D:52 C:20	D:21 C:30	(51)
Germany	D:150^*^ C:175^*^	D:6 C:8	(52)
Australia	D:60 C:21	D:40 C:38.1	(37)
Davis, CA, USA	194	D:14.4	(53)
Davis, CA, USA	245	C:9.4	(54)
Davis, CA, USA	334	D:15.5	(55)
Davis, CA, USA	132^*^	D:12.9	(27)
Ontario, Canada	93	D:52	(56)
Ontario, Canada	D:92 C:1	T:10.7	(57)
Ontario, Canada	102	D:58	(58)
Ontario, Canada	D:360 C:42	D:19 C:7.1	(59)
Corvallis, OR, USA	135	D:45	(60)
Ontario, Canada	139	D:10	(61)
Netherlands	D:116 C:115	D:25 C:15.7	(62)
Davis, CA, USA	273	C:0	(63)
Germany	D:165 C:135	D:5.5 C:3.7	(64)
Brazil	57	D:21.1	(65)
India	117	D:13.6	(66)
Iran	151	D:7.9	(67)
Flagstaff, AZ, USA	216	D:17.1	(18)
Japan	204	D:30	(68)
Spain	D:105 C:37	D:4.8 C:0	(19)
Knoxville, TN, USA	C:24	C:4.2	(46)
Brazil	82^*^	D:1.2	(69)
Spain	107	D:12.1	(70)
Spain	90^*^	D:6.7	(17)
Brazil	154	D:11.9	(71)
Germany	D:437 C:403	D:3.4 C:2.5	(72)
Eastern China	D:146 C:29	D:0.7 C:7	(28)
Brazil	C:304^*^	C:5	(25)

*D, Dog; C, Cat*.

* *Part of the sample cohort had diarrhea for the duration of the survey*.

*Comparison between dogs and cats to human (or other) were done, where both dog and cat totals were grouped. Therefore, no individual species prevalence was reported, rather a single total (T)*.

*C. difficile* strains are generally further classified based on the size variation in the 16s and 23s rRNA intergenic spacer region (Ribotype/RT). Most common human *C. difficile* isolates are RTs 106, 027, 078, 014, 002, and 020 (8, 13, 73–75). Of these, RTs 027 and 078 are generally referred to as hypervirulent strains and are associated with increased toxin production and outbreaks of severe CDI, and carry specific genomic characteristics (76, 77). Specifically, RT 027 is commonly associated with severe human CDI, predominantly in hospital settings (73, 78). This hypervirulent strain emerged and established a significant health problem in the last decade (73). Canadian, Spanish, and German studies identified CDI RTs 027, 078, and 014/0, all known causes of severe humans disease, in dogs (70, 72, 79). Human RT 106, becomes especially important due to its increasing prevalence and noted association with community-associated CDI in the United States and Europe (23, 24). RT 106 is also commonly isolated from dogs and cats (21, 25). Other ribotypes commonly isolated from dogs and cats worldwide include RT 039 in cats; RT 012 in dogs; and RTs 009, 010, and 014/20 overlapping between the two species (62, 64, 75, 80–82). *C. difficile* isolates from pets are often reported to be resistant to multiple antibiotics, including metronidazole (20, 23, 24, 70, 75, 82, 83). This poses a concern as a metrinidazole antibiotic-resistance adaptation can result in a recurrent CDI (rCDI), as observed in one human case (83). Ribotyping enables clinicians and researchers to quickly identify and predict potentially pathogenic strains of *C. difficile* that are isolated from clinical or environmental samples. However, *C. difficile* ribotyping may not be as sensitive as other methods of classification from an evolutionary or phylogenetic point of view, which will be discussed in later sections of this review.

## Prevalence of *C. difficile* in Other Companion Animal Species

The ubiquitous nature of *C. difficile* spores and their ability to stay in the environment for an extended period render several additional species of animals vulnerable to gut colonization and CDI via the feco-oral route. The organism has been isolated from healthy horses and exotic pets, with some strains more prevalent than others (11, 17, 21, 62, 84–87). Prevalence studies conducted in the Netherlands, Europe, and the Czech Republic demonstrated the presence of toxigenic and non-toxigenic strains of *C. difficile* in the horse gastrointestinal tract (62, 86, 87). A wide range of prevalence rates and diversity in *C. difficile* strains have been reported by these investigators. RTs 014 and 078 attracted special attention because they are also associated with human CDI outbreaks (62). Furthermore, multiple antibiotic resistance genes were found to be shared among both human and equine *C. difficile* isolates (87). As such, the genotypic similarities and overlap between human and equine CDI subtypes raise speculations on the possibility of interspecies transmission or adaptation of different toxigenic *C. difficile* strains (21, 86, 88).

Due to the limited number of studies conducted in exotic pets, information on toxigenic *C. difficile* in psittacine birds and small mammals (rabbits, ferrets, and rodents) is sparse (17). Recently, a novel non-toxigenic *C. difficile* ribotype was isolated from a pet reptile, indicating that exotic pets could carry uncommon *C. difficile* strains (17). Therefore, further studies are warranted to determine *C. difficile* prevalence and their zoonotic potential in less common household pets, including reptiles.

## Implications of Human-Pet Interactions in CDI Transmission

As asymptomatic carriers, household pets could potentially transmit pathogenic *C. difficile* strains to susceptible individuals such as the elderly and children, and could further disseminate CDI within a community (51, 89). A British research group investigated *C. difficile* colonization in infants and observed that a significant proportion of them (30–40%) were colonized with *C. difficile*, out of which 68% of the isolates were confirmed toxigenic (90). The results from this study pointed out a significant association between the colonization rate and presence of dogs in the household (90). A Canadian study revealed a 26% asymptomatic carriage rate in dogs that are in contact with individuals with CDI in households (91).

In 2006, a pathogenic human strain of *C. difficile* was identified in a dog that visited patients in a health care facility. Molecular characterization of the *C. difficile* isolate revealed that this service dog acquired the pathogen most likely from the health care facilities it visited (92). Therefore, an infected human can be considered as a route of initial *C. difficile* colonization in a susceptible pet. Studies have also demonstrated *C. difficile* colonization in dogs that participated in animal-assisted care programs in health care settings. Lefebvre et al. (93) observed that dogs visiting the health care facilities had a 2.4 times higher risk of acquiring *C. difficile* than those involved in other animal-assisted programs. In another study, dogs that had direct human contact, such as licking the patients or receiving treats were found to be at a greater risk of acquiring *C. difficile* (94). These interactions suggest that CDI may be perpetuated within the community. In a more recent study, spores of toxigenic *C. difficile* were identified in the nasal secretion of pet dogs adding to the risk of direct transmission of this bacteria to humans in close contact (95). A study conducted in Spain identified toxigenic *C. difficile* isolates in playground sandboxes that are unprotected from dogs, posing an additional public health risk to a vulnerable young population (96). Additionally, mechanical spread of *C. difficile* from houses to the community through shoe soles and dog paws have been reported (97).

Recurrence of CDI usually occurs in ~20% of individuals within a month after primary treatment (98). However, a definitive cause of rCDI and a radical method for preventing this recurrence remains unknown. rCDI can be a result of relapse with the same strain or infection with another *C. difficile* strain (99). Thus, *C. difficile* transmission between pets and susceptible humans should be considered as one of the possible mechanisms of reinfection in rCDI. As an example, RT 106, commonly found in dogs and cats, has shown to cause a higher recurrence rate in humans as opposed to more virulent strains (24). A possible explanation for this phenomenon could be the reported higher sporulation rate of RT 106, which can increase the chance of reinfection from contaminated surfaces or the retention of spores in the gut (100). However, a higher recurrence rate of this ribotype can also be potentially attributed to the presence of silent carriers of infection, e.g., pets in the household which can harbor, shed, and transmit RT 106 to the patient.

Isolation and molecular typing of *C. difficile* from rCDI patients are crucial in determining the potential origin of rCDI strains but such data are scanty in the literature. A limited investigation conducted in Minnesota, United States, identified *C. difficile-*positive humans in homes with pets, where the owner had experienced a previous episode of CDI (101). It was unclear whether the human *C. difficile* colonization resulted from the previous human CDI or exclusively transmitted from pet and household surfaces. Additionally, the number of households with pets in this study was too small to further examine pets as a valid source (101). As such, owners should be advised to take extra precautions when clostridial diarrhea in their pets, especially in consideration of CDI recurrence.

Although the interspecies transmission of *C. difficile* between dogs and humans appears to be a legitimate concern, there is a contrasting but beneficial aspect of human-pet interaction for those patients suffering from CDI. Studies have demonstrated that dogs can be trained to detect *C. difficile* infection at the initial stage of clinical disease and in patients experiencing non-specific symptoms (102–104). A few small scale studies even report a potential protective effect of pet ownership in rCDI (105). However, precautions must still be taken to minimize the risk of further spread of CDI outside of health care facilities through human-pet interactions until the most accurate association is elucidated.

## Molecular Epidemiology, Phylogeny, and Potential Interhost Adaptation of Pet *C. difficile*

Detailed comparative genome-wide characterization of pet *C. difficile* isolates is required to determine transmission between pets and humans within a household or in a wider environment. Sequence-based genotyping techniques such as Multiple-Locus Variable number tandem repeat Analysis (MLVA), Multilocus Sequence Typing (MLST), Core-genome MLST, or whole-genome Single Nucleotide Polymorphisms (SNP) are based on the changes that occur in conserved parts of the *C. difficile* genome, which adapts minimally in the course of evolution. Specifically, methods such as maximum likelihood estimations help calculate the length of a branch in a phylogenetic tree and predict the probable evolutionary rates (106). Maximum likelihood analysis conducted on a large database, pubMLST, groups *C. difficile* isolates diversity into five major distinct clades: clade 1–5 (107). There are three additional cryptic clades, C-I to C-III, which comprise of strains not included in the five major clades (108, 109). Clades are further subcategorized into multiple multilocus Sequence Types (ST) of *C. difficile* within which different RTs are grouped. Clade 1 has the most diverse STs among all clades, comprised of the most frequent pet associated non-hypervirulent STs. Clade 2 is composed of STs 1, 32, and 67. ST 1 includes the human hypervirulent strain RT 027. A notable member of clade 5 is ST 11, under which the emerging human hypervirulent strain RT 078 is grouped. This RT is widely isolated from food animal species (110). MLST analysis conducted on dog strains isolated in Arizona, United States, demonstrated that several sequence types belong to clade 1 (18). Among these STs, there was a higher frequency of STs 2, 3, 42, and 15. The former three are also observed in equivalent levels in humans (18). Although RTs 027 and 078 are rarely isolated from pets, more general sequence types appear to be shared between dogs and humans, which suggests possible sharing of virulent *C. difficile* strains.

Although MLVA, MLST, and SNP genotyping techniques are ideal in establishing genetic distance and relatedness, they are less useful in providing information on the unique qualities of individual isolates, such as antibiotic resistance genes, pathogenicity loci, transposons, and mobile elements. Therefore, it is important to study the hypervariable regions of the *C. difficile* genome from pets, where the acquisition and loss of genetic material can occur, particularly that which may facilitate the rapid adaptation of bacteria in a new environment or host. Such genome-wide characterization can provide this information and other unique features of a given *C. difficile* isolate and help fill the current large knowledge gap.

Identification of human-specific and pet-specific genes could be used as markers of intermixing of *C. difficile* genetic material to understand host-specific elements that could potentially alter the virulence capacity of *C. difficile* STs in pets. In 2009, Stabler et al. conducted a study to understand the mechanism of the emergence of human epidemic and hypervirulent *C. difficile* RT 027 strain. The authors compared the genome of hypervirulent RT 027 to a non-epidemic RT 027 (CD196) identified in very isolated incidents, and *C. difficile* RT 012 (CD630; the reference genome). The comparative genomic analysis identified a number of recently acquired genetic elements encoding a unique phage island, two-component regulatory systems, and transcription regulators exclusive to the epidemic “hypervirulent” RT 027 strain and the possible cause of its emergence (111). Such an analysis in pet *C. difficile*, in combination with that of their respective owners, could help predict the possible emergence of *C. difficile* strains of public health concern.

Understanding genome-wide changes is essential for identifying host-specific adaptation in *C. difficile*. Within the conserved (core) genome, toxigenic *C. difficile* encodes for a 19.6-kb Pathogenicity Locus (PaLoc), which constitutes toxins genes (*tcdA* and *tcdB*), regulatory genes (*tcdC, tcdR)*, and a holin-like gene (*tcdE*) responsible for toxin secretion. In contrast, non-toxigenic strains do not exhibit this length of sequence anywhere in their genome (112). Interestingly, non-toxigenic *C. difficile* strains have acquired toxin production by horizontal gene transfer of the PaLoc (113). Furthermore, a closely related pathogen, *C. perfringens*, was also found to gain virulence by way of horizontal gene transfer in the gut environment (114). This phenomenon points out the possibility of an alternate mechanism for the emergence of zoonotic *C. difficile* strains resulting in the intermixing of pet and human *C. difficile* strains. Furthermore, polymorphisms and deletions exist within the PaLoc that may affect the levels, types, and variants of one or both toxins (115, 116). As the PaLoc is indispensable in CDI pathogenesis, understanding the changes within the PaLoc region of pet and human *C. difficile* isolates can be useful for predicting the emergence of a hypervirulent and highly toxigenic *C. difficile* strains.

## Conclusion

*Clostridioides difficile* infection is becoming a significant public health concern as the disease severity, and the proportion of individuals infected in community settings is steadily increasing. Studies from various parts of the world suggest household pets as carriers and potential sources for pathogenic *C. difficile* to humans. Detection of similar *C. difficile* isolates from companion animals and humans suggest potential pet-borne transmission of community-associated CDI. However, large scale prevalence studies among pet and owner pairs, with whole-genome characterization of pet and human *C. difficile* isolates, are necessary to understand host-specific genomic elements, mobile genetic elements, antibiotic resistance genes, and inter- and intra- sequence type variations. Such studies are necessary to predict an already occurring or impending emergence of zoonotic *C. difficile* strains. Unfortunately, most of the available studies in the literature are conducted on a small scale with limited investigations on genomic details of pet *C. difficile* isolates. Additionally, systematic studies on *C. difficile* carriage in cats are limited, even with the potential risks posed by cat litter boxes. Similarly, systematic studies on *C. difficile* carriage in owner-pet pairs in a household are limited. Therefore, further studies, routine health screening of companion animals and owners for *C. difficile* carriage, and genomic characterization of pet *C. difficile* isolates are warranted to address this knowledge gap.

## Author Contributions

SM conceptualized the idea. SM and CT designed the project outline. BH, CT, and AV conducted the literature search and analysis. CT, BH, SM, and AV wrote the manuscript. BS reviewed and edited the manuscript. All authors contributed to the article and approved the submitted version.

## Conflict of Interest

The authors declare that the research was conducted in the absence of any commercial or financial relationships that could be construed as a potential conflict of interest.
